# Challenges facing a new prehospital care service in the developing world: the Nepali Ambulance Service (NAS)

**DOI:** 10.1186/1757-7241-21-S1-S13

**Published:** 2013-05-28

**Authors:** N Wilson, A Cunningham, E Coleman, C Patterson, H Nnajiuba, A Guerrero, D Frith

**Affiliations:** 1 Trauma Sciences, Queen Mary University of London, London, UK; 2 InterTrauma Medical Consulting, New York City, USA

## Introduction

Prehospital trauma systems have been shown to significantly reduce mortality in resource limited areas[1]. Nepal has an annual per-capita health expenditure of $30[2]. In 2012, the first professionally trained, non profit Nepali Ambulance Service (NAS) began operations with five ambulances. It is a non-profit initiative in Kathmandu valley aiming to deliver rapid ambulance transport and medical care for all patients.

This case study aims to document the challenges experienced by the directors and technicians responsible for implementing this novel service, with a view to sharing their experiences with organisations developing pre-hospital care systems in similar regions.

## Methods

A UK doctor and a camera technician travelled to Nepal in June 2012. Recorded interviews were conducted with the NAS medical director (MD), chief executive officer (CEO) and two emergency medical technicians (EMT1/2). Interviews were performed in an open question fashion using G3 EW100 radio microphones (*Seinnheiser*). The content was transcribed, analysed and subdivided into themes.

## Results

Over two hours of discussion was recorded forming a 9000 word transcript. Problematic themes are identified below with examples of supporting statements.

Financial – “The biggest hurdle still for us is the funding.” CEO

Bureaucratic – “We had to wait a year while our ambulances were stuck in customs.” CEO

Infrastructure – “In Nepal there are no road names, no road systems and no house numbers.” CEO

Logistical – “Every month there will be some kind of strike. So often we cannot fill our petrol tank.” EMT2

Cultural - “The knowledge that professional help in the ambulance on the way to hospital is important is lacking in our public.” MD

## Discussion

With road traffic incidents to become the fifth leading cause of death worldwide the UN has prioritised establishing prehospital care in every nation[3]. This study has identified numerous barriers that can be overcome when starting a developing world ambulance service. Understanding their progress will enable others to anticipate obstacles and develop more effective methods for overcoming them.
